# Effectiveness and Safety Analysis of PIs/r Based Dual Therapy in Treatment-Naïve, HIV/AIDS Patients: A Network Meta Analysis of Randomized Controlled Trials

**DOI:** 10.3389/fphar.2022.811357

**Published:** 2022-03-04

**Authors:** Liu Hui, Han Xiaoxu, Wang Yuqi, Wang Peng, Wang Xin, Yi Yunyun, Li Xin

**Affiliations:** Department of Center of Integrated Traditional Chinese and Western Medicine, Beijing Ditan Hospital, Capital Medical University, Beijing, China

**Keywords:** aids, PIs/r, dual therapy, triple therapy, network meta-analysis, HIV infection

## Abstract

**Background:** Dual anti-retroviral therapy is the main proven valuable intervention type for treating naïve HIV/AIDS. Currently, no high-quality evidence is available regarding the best dual schemes.

**Objectives:** The aim of this study is to evaluate the effectiveness and safety of PIs/r-based dual therapy in treatment-naïve HIV/AIDS patients by using network meta-analysis.

**Methods:** Randomized controlled trials of PIs/r-based dual therapy in treatment-naïve HIV/AIDS were searched based on Embase, PubMed and Cochrane library database from January 2006 to June 2021. Taking viral suppression rate, CD4^+^T cell count changes from baseline as the primary indicator and adverse events rate as secondary indicator, the network meta-analysis was performed on Review Manager and STATA software. Heterogeneity was assessed by the Q statistic and I^2^. We registered our protocol in Prospero with ID CRD42021275466.

**Results:** Among 15 randomized controlled trials (3,497 patients and 7 PIs/r-based dual therapy) were reviewed in this study. According to the forest map, DRV/r + INSTIs was more effective compared to triple therapy (TT) in viral suppression [OR 0.82, 95% CI (0.61–1.11)], in CD4^+^T cell count changes from baseline [MD 1.9, 95% CI (0.7, 3.1), *I* ^*2*^ 86%], in adverse events [OR 0.98, 95% CI (0.68–1.39)]. Furthermore, SUCRA ranking analysis indicated that DRV/r + INSTIs was superior to TT in viral suppression (DRV/r + INSTIs 75.5% > TT 41.2%) and in immune construction (DRV/r + INSTIs 67% > TT 42%). In addition, DRV/r + INSTIs was similar to TT in adverse events (DRV/r + INSTIs 54.9% ≈ TT 54.7%).

**Conclusion:** DRV/r + INSTIs was obviously superior to TT in viral suppression and immune reconstruction, and was not higher than TT in adverse events.

**Systematic Review Registration:** https://www.crd.york.ac.uk/prospero/, identifier CRD42021275466

## Introduction

Antiviral treatment significantly reduces the mortality of HIV/AIDS and greatly prolongs their life expectancy. Since 1996, HAART has become the standard treatment for AIDS, and more than 90% person of HIV/AIDS have received this treatment (Garcia-Tejedor et al., 2009). In the recent years, researchers explored constantly the selection and collocation of HAART drugs in order to avoid or reduce adverse reactions, raise patients’ medical compliance and improve the life quality of HIV/AIDS (Batchelder et al., 2013). Although the current antiviral scheme is continuously optimized and well tolerated, there needs long-term or even lifelong medication, which will inevitably bring patients’ economic burden and some adverse events (Carr and Amin, 2009). A study by Achhra et al. (Achhra et al., 2016) showed that the combination of TDF + FTC caused kidney damage and bone changes. The association of EFV+2NRTIs brought some damage on nerve, which predictively affects the long-term management of AIDS to make some patients fail in antiviral treatment due to drug leakage and withdrawal. Therefore, there are many simplified treatment schemes emerged to improve patient compliance and to reduce medical costs in current study, including dual therapy, monotherapy and intermittent treatment et al. (de Miguel Buckley et al., 2018; Rossetti et al., 2018). Some statistical analysis provided by Di Carlo et al. (Di Carlo et al., 2021) showed that DTG-based dual simplified therapy had better effectiveness and similar safety compared to TT in those patients who has baseline viral load > 10^5^/ml or CD4^+^T ≤ 200 cells/ul. Filippo et al. (Del Puente et al., 2020) found that RAL-based dual simplified regimen was not inferior to triple regimen in inhibiting viral load, and had a better role in helping immune reconstruction. These studies emphasize the drawbacks of traditional antiviral therapy.

All major guidelines suggested that the first-line ART regimen was composed of Integrase Inhibitors (INSTIs) + one or two Nucleoside/Nucleotide Reverse Transcriptase Inhibitors (NRTIs). European AIDS Clinical Society (EACS) Guidelines (Blanco et al., 2018) recommended that Ritonavir-boosted Protease Inhibitors (PIs)/r was considered as a core drug to combine with 2NRTIs. PIs/r inhibits virus replication by inhibiting proteolytic activity, preventing the cleavage of HIV pro-protein and forming mature infectious virus particles. At present, LPV/r and DRV/r belong to the PIs category. Some scholars (Pisaturo et al., 2020) proposed that PIs/r-based DT plays an important role in inhibiting HIV. A randomized controlled trials (RCTs) (Reynes et al., 2013) found that LPV/r + RAL was not inferior to traditional TT in inhibiting HIV, and was lower in the occurrence of adverse events. Di Cristo V et al. (Di Cristo et al., 2020) showed that DRV/r + RAL had great advantages in immune reconstruction by comparing with the traditional TT. Although more and more clinical studies have proved that the PIs/r-based dual-simplified therapy is an effective treatment, there is unable to clarify the comparison between various PIs/r-based dual-simplified schemes and TT.

Network meta-analysis (also called mixed-treatment comparison) is an extension of traditional meta-analysis based on indirect comparison or combining results of indirect comparison and direct comparison. It combines clinical evidence of direct comparison and indirect comparison, and ranks the efficacy of different therapy schemes (Buti et al., 2011). Therefore, in this study, both the effectiveness and safety of PIs-based dual schemes for treatment-naïve HIV/AIDS patients are analyzed by network meta-analysis, which can provide clinical medication evidence to HIV/AIDS.

## Materials and Methods

### Data Sources and Literature Quality

Two researchers (Liu Hui and Han Xiaoxu) searched to original reports using the Embase, PubMed and the Cochrane library from January 2006 to June 2021, using both medical subject heading (MeSH) terminology and relevant keywords to identify articles that analyzed the effectiveness and safety of dual versus triple antiretroviral therapy in HIV-naïve patients. Search terms include: “HIV” (or “acquired immunodeficiency syndrome”), “antiretroviral” (“protein inhibitors” or “PIs”), “randomized clinical trials” (or “RCTs”). We made a protocol before commencing the study and registered it on the PROSPERO International Prospective Register (CRD42021275466).

### Study Selection

All studies in this meta-analysis had to meet the following inclusion criteria: 1) RCTs; 2) Treatment-naïve, HIV/AIDS patients who are greater than 18 years old; 3) Intervention measures include dual simplified schemes and triple schemes based on PIs/r; 4) One or more of the following outcomes are assessed: the number of HIV suppression cases, the change value of CD4^+^T cell count from baseline and the number of adverse events.

Exclusion criteria: 1) Research types: summary, abstract, letter, conference or report; 2) The researches about experienced HIV/AIDS or non-HIV/AIDS patients; 3) Comparative study without PIs/r drugs; 4) Primitive study related to the efficacy and adverse events of only one drug.

### Literature Data Extraction

Data extraction is separately recorded by two researchers. If there is any inconsistency in the recorded information, the third researcher will be consulted to solve this difference. The basic information included in this study: the first author, the year of publication, country, the intervention measures, the cases of each intervention group, the age and the follow-up time (weeks). The outcome indicators of naïve HIV/AIDS patients include viral suppression rate, △CD4^+^T cell count from baseline and adverse events rate.

### Quality Assessment

The RCT Cochrane Risk of Bias Tool (version 5.0) was used to evaluate the quality of the included studies, including: 1) allocation concealment, 2) randomization method, 3) blind method, 4) integrity of outcome data, 4) selective report results, and 5) other biases. The Review Manager 5.4 software provided by Cochrane Collaboration network was used to draw the literature quality evaluation chart, which aims to evaluate the bias of the research directly.

### Statistical Analysis

Before the analysis, we rechecked the extracted data. Firstly, the network meta-analysis for the extracted data was performed by using Review Manager 5.3 software. Both Odds Ratio (OR) and 95% Confidence Interval (95% CI) were used for discontinuous variable analysis, and means/SD was used for continuous variable analysis. Effect sizes were synthesized using a fixed-effect model except when there was significant heterogeneity, for which the random-effects model was used. Statistical heterogeneity was considered significant when *I* ^*2*^ > 50%, *p*-value < 0.05. Subsequently, STATA (version 15) software was used to draw the network meta-analysis evidence network plot, funnel plot, consistency test plot, ranking plots of the extracted data. Interestingly, the SUCRA package provided by STATA software was not only used to obtain the ranking results of each intervention measure, but also calculate the Area Under the Curve (AUC) to draw the cumulative probability ranking diagram. Furthermore, the outcome indicators were clustered based on the SUCRA value, which can provide evidence to screen the best DT between the two clustering indicators.

## Results

### Study Selection

**FIGURE 1:**
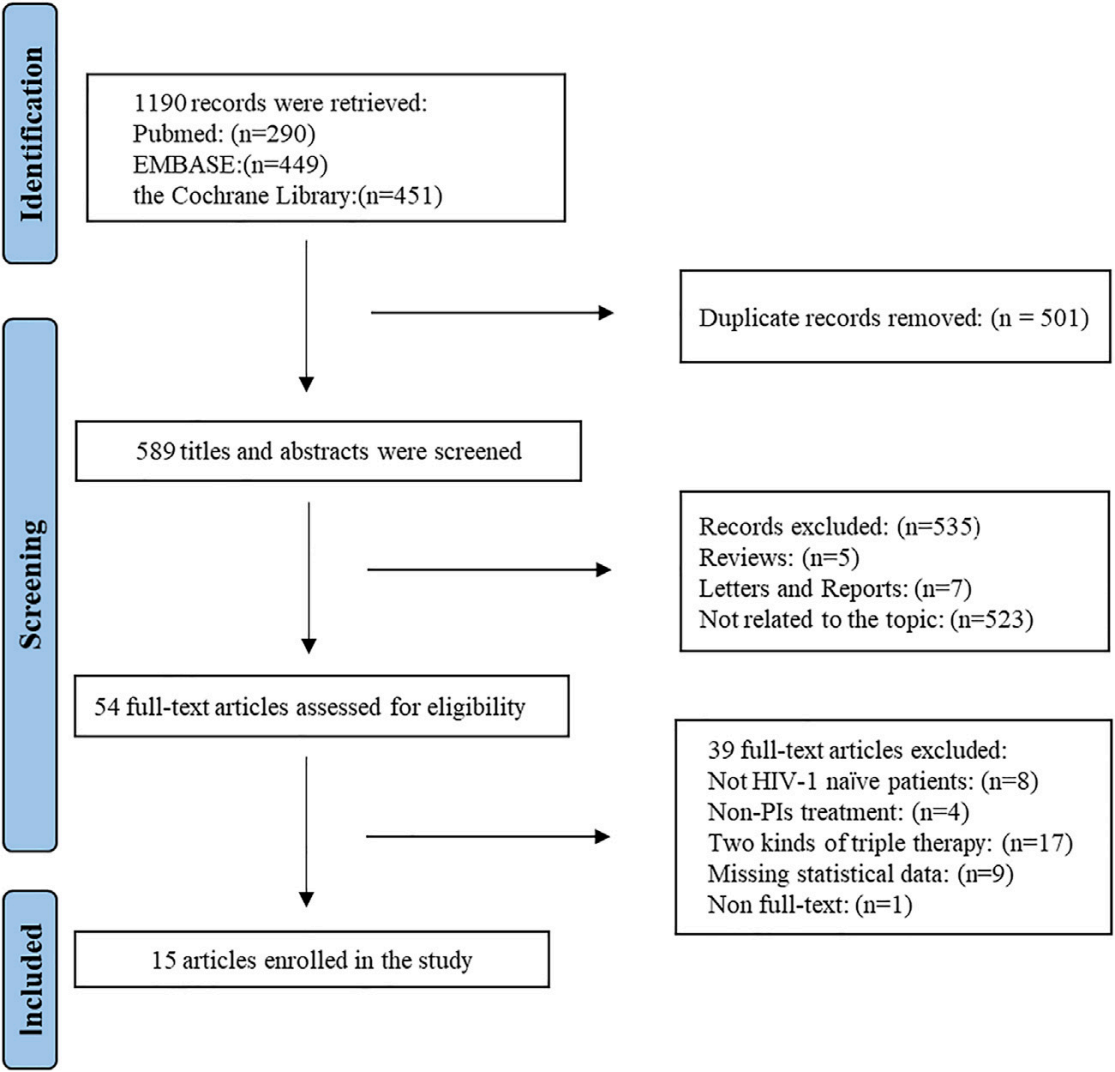
PRISMA flow chart of study selection.

A flow chart describing the identification and selection of the articles included is shown in Figure 1. A total of 1,190 studies were identified from the search of electronic database. All of studies were imported into Endnote X8 software. Of these, 501 duplicate articles were eliminated and 636 articles were excluded on the base of title and abstract. After reading the full text, it was shown that 76 articles were excluded: eight articles whose research objects were not naïve treated HIV/AIDS patients, 41 articles whose intervention measures were non-PIs/r treatment, 17 articles which compared the two kinds of TT, nine articles which lacked statistical data and 1 article which did not obtain the full text. Finally, 15 studies were obtained (Cameron et al., 2007; Van Vonderen et al., 2009; Yeni et al., 2009; Pinola et al., 2010; Reynes et al., 2011; Ulbricht et al., 2011; Reynes et al., 2013; Bedimo et al., 2014; Cahn et al., 2014; Raffi et al., 2014; Nozza et al., 2015; Cook et al., 2016; Stellbrink et al., 2016; Winston et al., 2017; Stella-Ascariz et al., 2018).

### Study Characteristics

The main characteristics of 15 studies included in the meta-analysis are summarized (Supplementary Material Table S1). Research features: 15 studies were RCT, which were published from 2007 to 2018, and the follow-up time was 12–96 weeks. Characteristics of subjects: The subjects are mainly young and middle-aged, and there are more males than females. The baseline level of CD4^+^T cells is between 120 and 348 cells/mm^3^. Characteristics of intervention measures: two studies directly compared LPV/r + PIs and TT; two studies directly compared LPV/r + NRTIs and TT; two studies directly compared LPV/r + INSTIs and TT; two studies directly compared LPV/r + CCR5 and TT; five studies directly compared DRV/r + INSTIs and TT; two studies directly compared LPV/r + NNRTIs with TT, DRV/r + CCR5 with TT.

### Quality Assessment

**FIGURE 2:**
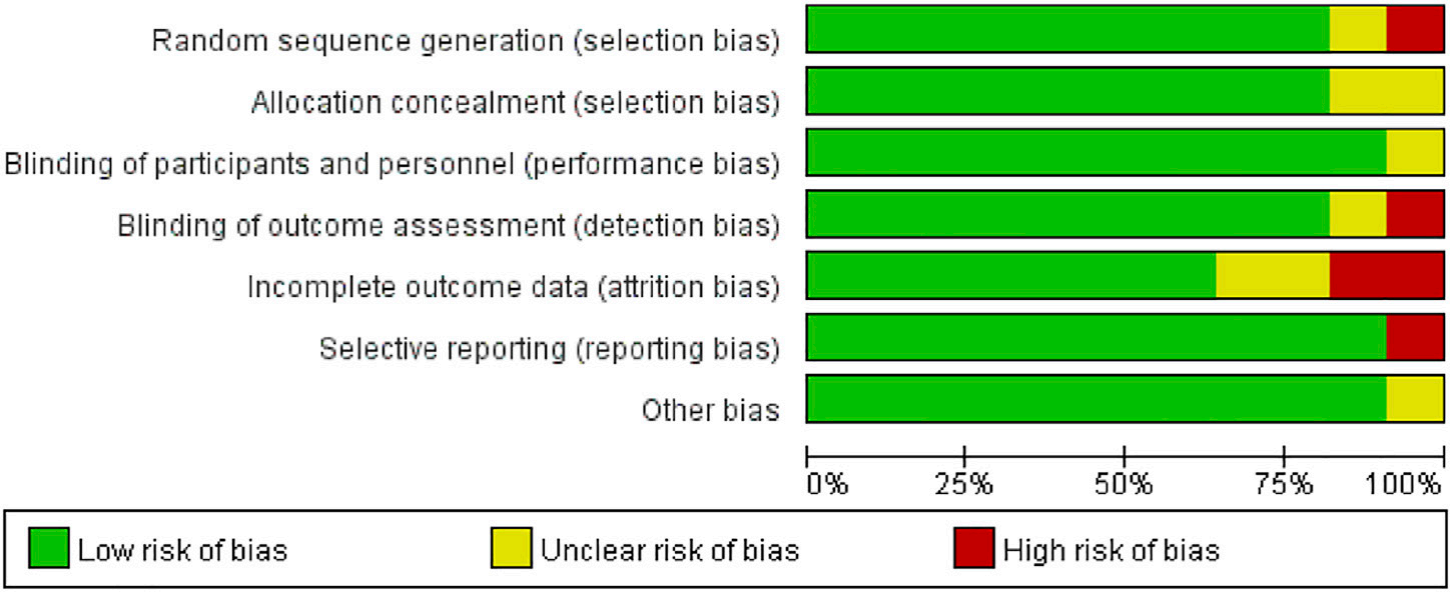
Risk of bias assesment summary.

The summary of study quality evaluation is shown in Figure 2. About 10% of the studies had uncertainty in random sequence allocation, so there may be allocation bias in random allocation. About 18% of the research reported that it is uncertain in the measurement of outcome indicators, which exhibits unknown bias in the outcome indicators probably. About 24% of the results report was incomplete, which means some distribution bias in the completeness of the results report. About 10% of the results were reported selectively, so there may be report bias. In the overall quality of literature, all included RCTs were rated to be low or moderate risk and there was no study rejected because of low quality.

### Outcomes

#### Viral Suppression Rate

**FIGURE 3:**
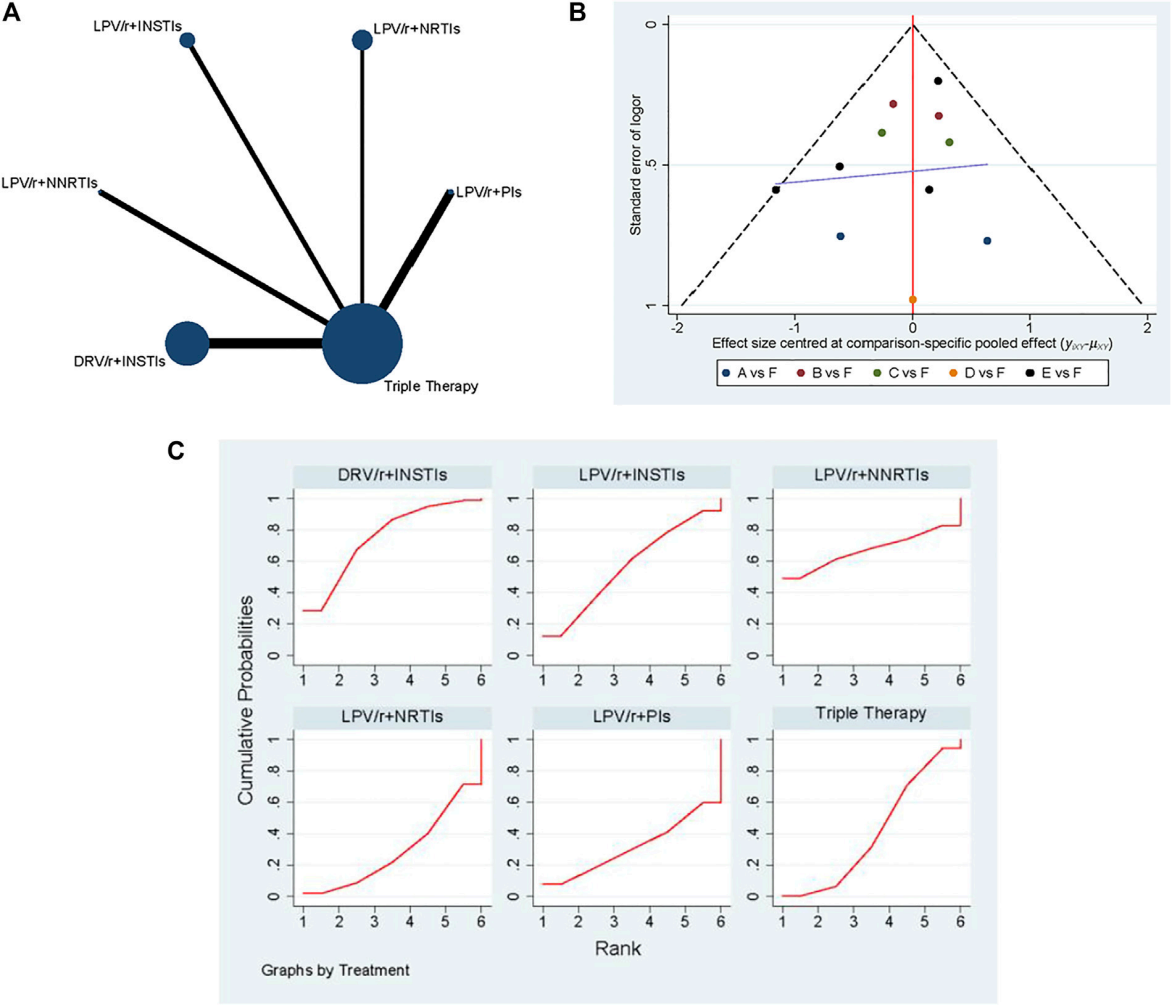
The results of network meta-analysis with viral suppression rate. **(A)** The network diagram. **(B)** The comparison-correction funnel diagram (A: LPV/r + PIs, B: LPV/r + NRTIs, C: LPV/r + INSTIs, D: LPV/r + NNRTIs, E: DRV/r + INSTIs, F: TT). **(C)**: SUCRA value ranking chart.

A total of 11 studies have collected the indicators of viral suppression rate, and the network diagram is shown in Figure 3A. The most patients were treated with DRV/r + INSTIs, and the most studies directly compared DRV/r + INSTIs with TT. The comparison-correction funnel diagram is shown in Figure 3B. The direct comparison of two different interventions were indicated by the dots with different colors. The funnel diagram distribution was basically symmetrical. In addition, there was no closed loop among the interventions, and it was not necessary for consistency test.

**FIGURE 4:**
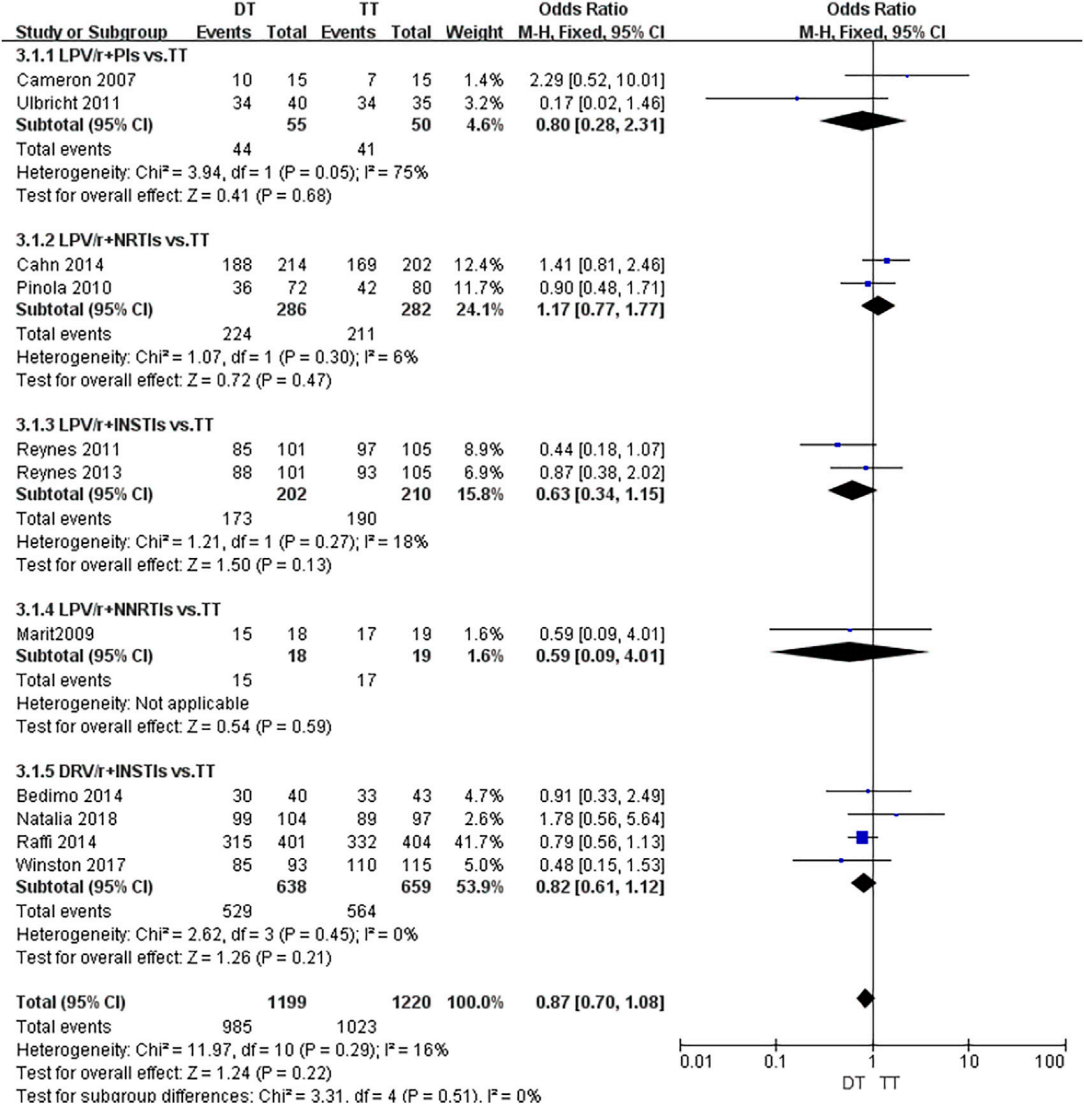
Node-splitting of network meta-analysis based on viral suppression.

As shown in Figure 4, the fixed effect model was used due to the presence of heterogeneous with *I* ^*2*^ = 16%. Although it was suggested that there was no significant statistical difference in viral suppression between DT and TT [*OR* 0.87; 95% *CI* (0.7–1.08)], the OR was tendency to DT. According to the concrete analysis of different dual schemes, DRV/r + INSTIs [*OR* 0.82; 95% *CI* (0.61–1.12)], LPV/r + INSTIs [*OR* 0.63; 95% *CI* (0.34–1.15)] was superior to TT.

Consequently, SUCRA value was used to sort different schemes in viral suppression rate (Figure 3C). It held with the following relationship: DRV/r + INSTIs (75.5%) > LPV/r + NNSTIs (67.4%) > LPV/r + INSTIs (56.4%) > TT (41.2%) > LPV/r + PIs (31.1%) > LPV/r + NRTIs (28.4%). In all, we drawn the conclusion that DRV/r + INSTIs and LPV/r + INSTIs were superior to TT in viral suppression.

#### △CD4^+^T Cell Counts From Baseline

CD4^+^T cell counts is an indicator related to immune reconstruction. A total of nine articles referred to the number of CD4^+^T cells change from baseline, the network diagram and comparison-correction funnel diagram was shown in Supplementary Material Figures S1A, B. Heterogeneity analysis suggested that there was obvious heterogeneity among the research results (*I* ^*2*^ = 76%, *p* < 0.001, Supplementary Material Figure S2). Randomized effect model was used for analysis [MD 1.91; 95% *CI* (0.71–3.11)] and the overall effect test was applied (*Z* = 3.12, *p* = 0.002). According to the concrete analysis of different dual schemes, it was shown that LPV/r + PIs (*MD* −43.69, 95% *CI* (−95.69, 8.31), *I* ^*2*^ = 56%), LPV/r + NNRTIs (*MD* −30, 95% *CI* [−130.79, 70.79)], DRV/r + INSTIS [*MD* 1.9, 95%*CI* (0.7, 3.1), *I* ^*2*^ = 86%] were superior to TT. The following relationship was got by SUCRA value (Supplementary Material Figure S1C): LPV/r + PIs (81.9%) > DRV/r + INSTIs (67%) > LPV/r + NNRTIs (65.7%) > TT (42%) > LPV/r + NRTIs (39%) > LPV/r + CCR5 (4.4%). Therefore, LPV/r + PIs, DRV/r + INSTIs and LPV/r + NNRTIs were all superior to TT in improving immune reconstruction, which was consistent with the results of forest map.

#### Adverse Events

A total of 10 studies have counted the incidence of adverse events. The network diagram and comparison-correction funnel diagram were shown in Supplementary Material Figures S3A, B. Heterogeneity analysis suggested that there was obvious heterogeneity among the research results (*I* ^*2*^ = 56% *p* = 0.02, Supplementary Material Figure S4). Randomized effect model was used for analysis [*OR* 0.93, 95% *CI* (0.77–1.12)] and the overall effect test was applied (*Z* = 0.75, *p* = 0.46). According to the concrete analysis of different dual schemes, it was shown that there was no significant difference in the possibility of adverse events among DRV/r + INSTIS (*OR* 0.98, 95%*CI* [0.68–1.39]), DRV/r + CCR5 [*OR* 0.99, 95% *CI* (0.61–1.6)] and TT. The following relationship was got by SUCRA value (Supplementary Material Figure S3C): LPV/r + NRTIs (95.6%) > LPV/r + INSTIs (61.8%) > DRV/r + INSTIs (54.9%) > TT (54.7%) > LPV/r + CCR5 (51.2%) > LPV/r + PIs (30.5%) > LPV/r + NNRTIs (1.2%). In all, the possibility of adverse events in LPV/r + PIs and LPV/r + CCR5 was lower than that in TT, and there was no significant difference between DRV/r + INSTIs and TT.

### The Results of Network Meta-Analysis on HIV Inhibition and Adverse Events

In Table 2, it was shown that the result of meta-analysis was no significant difference in HIV inhibition between different dual schemes (*p* > 0.05), but the possibility of adverse events in DRV/r + INSTIs scheme was lower than that in LPV/r + NRTIs [*OR* 0.19, 95% *CI* (0.08, 0.44)], LPV/r + INSTIs [*OR* 0.28, 95% *CI* (0.12, 0.66)], LPV/r + CCR5 [*OR* 3.43, 95% *CI* (1.48, 7.94)], DRV/r + CCR5 [*OR* 3.35, 95% *CI* (1.57, 7.16)] and TT (*OR* 3.39, 95% *CI* (1.38, 8.36)], respectively.

**TABLE 1:** Study and patient characteristics of included studies.

Author/Year/Country	Intervention	Cases	Follow-up (Weeks)	△CD4^+^T (means ± SD)	Viral suppression (%)	AEs (%)
Cameron/2007/Canada	LPV/r + SQV	15	48	93 ± 75.6	63	38
	LPV/r + ZDV/3TC	15	48	163 ± 97.1	50	57
Pinola/2010/Italy	LPV/r + TDF	72	72	—	51.4	84.7
	LPV/r+2 NRTIs	80	72	—	52.5	83.8
Reynes/2011/France	LPV/r + RAL	101	48	—	84.5	27.7
	LPV/r + TDF/FTC	105	48	—	93.8	27.6
Ulbricht/2011/Germany	LPV/r+3TC/ZDV	35	48	142 ± 146.5	100	28.5
	LPV/r + ATV	40	48	125.1 ± 250.2	86	45
Reynes/2013/France	LPV/r + RAL	101	96	—	88.9	30.7
	LPV/r + TDF/FTC	105	96	—	85.2	34.3
Bedimo/2014/American	DRV/r + RAL	40	48	167 ± 119.3	75	12.5
	DRV/r + TDF/FTC	43	48	207 ± 185.2	76.7	5
Cahn/2014/Mexico	LPV/r+3TC	214	48	227 ± 159.3	88	—
	LPV/r+3TC/FTC	202	48	217 ± 169.5	84	—
Raffi/2014/UK	DRV/r + RAL	401	96	268 ± 9.183	78.6	18.2
	DRV/r + TDF/FTC	404	96	266 ± 8.163	82.2	18.5
Nozza/2015/Italy	LPV/r + MVC	26	48	286 ± 118.5	—	—
	LPV/r + TDF/FTC	24	48	199 ± 118.5	—	—
Winston/2017/UK	DRV/r + RAL	93	96	—	92	—
	DRV/r + TDF/FTC	115	96	—	96	—
Natalia/2018/Spain	DRV/r + RAL	104	96	265.52 ± 159.64	95.2	—
	DRV/r + TDF/FTC	97	96	253.4 ± 167.43	91.8	—
Marit/2009/Amsterdam	LPV/r + NVP	18	96	240 ± 185.2	83	—
	LPV/r + ZDV/3TC	19	96	302 ± 118.5	89	—
Yeni/2009/France	APL200 mg + LPV/r	54	12	—	50	78
	APL400 mg + LPV/r	55	12	—	48	-
	APL800 mg + LPV/r	56	12	—	54	-
	3TC/ZDV + LPV/r	26	12	—	75	50
Paul/2016/Carolina	RAL + DRV/r	20	48	206 ± 52.61	—	—
	EFV/FTC/TDF	20	48	279 ± 55.6	—	—
Stellbrink/2016/Germany	MVC + DRV/r	396	96	—	—	90.9
	TDF/FTC + DRV/r	401	96	—	—	91

*Adverse Events: AEs.

**TABLE 2:** Cell Counts from Baseline Meta-analysis Results of Viral Suppression Rate (upper right corner) and Adverse Events (lower left corner) (*OR* value and 95%*CI*).

LPV/r + PIs	0.95 (0.26,3.51)	0.68 (0.17,2.64)	0.54 (0.15,1.96)	—	0.81 (0.26,2.55)	-	0.47 (0.05,4.88)
2.20 (0.91,5.31)	**LPV/r + NRTIs**	0.72 (0.28,1.85)	0.57 (0.24,1.34)	—	0.85 (0.46,1.58)	-	0.50 (0.06,4.16)
1.51 (0.61,3.74)	0.69 (0.39,1.20)	**LPV/r + INSTIs**	0.79 (0.31,2.01)	—	1.19 (0.58,2.44)	-	0.70 (0.08,5.99)
0.42 (0.14,1.26)	0.19 (0.08,0.44)	0.28 (0.12,0.66)	**DRV/r + INSTIs**	—	1.50 (0.83,2.71)	-	1.13 (0.14,9.32)
1.43 (0.59,3.43)	0.65 (0.39,1.08)	0.94 (0.54,1.64)	3.43 (1.48,7.94)	**LPV/r + CCR5**	—	—	—
1.41 (0.55,3.60)	0.64 (0.35,1.18)	0.93 (0.49,1.77)	3.39 (1.38,8.36)	0.99 (0.54,1.81)	**TT**	—	1.70 (0.22,12.88)
1.39 (0.62,3.10)	0.63 (0.44,0.91)	0.92 (0.60,1.40)	3.35 (1.56,7.16)	0.98 (0.68,1.39)	0.99 (0.61,1.60)	**DRV/r + CCR5**	—
—	—	—	—	—	—	—	**LPV/r + NNRTIs**

### Cluster Diagram of Pairwise Outcome Indicators

**FIGURE 5:**
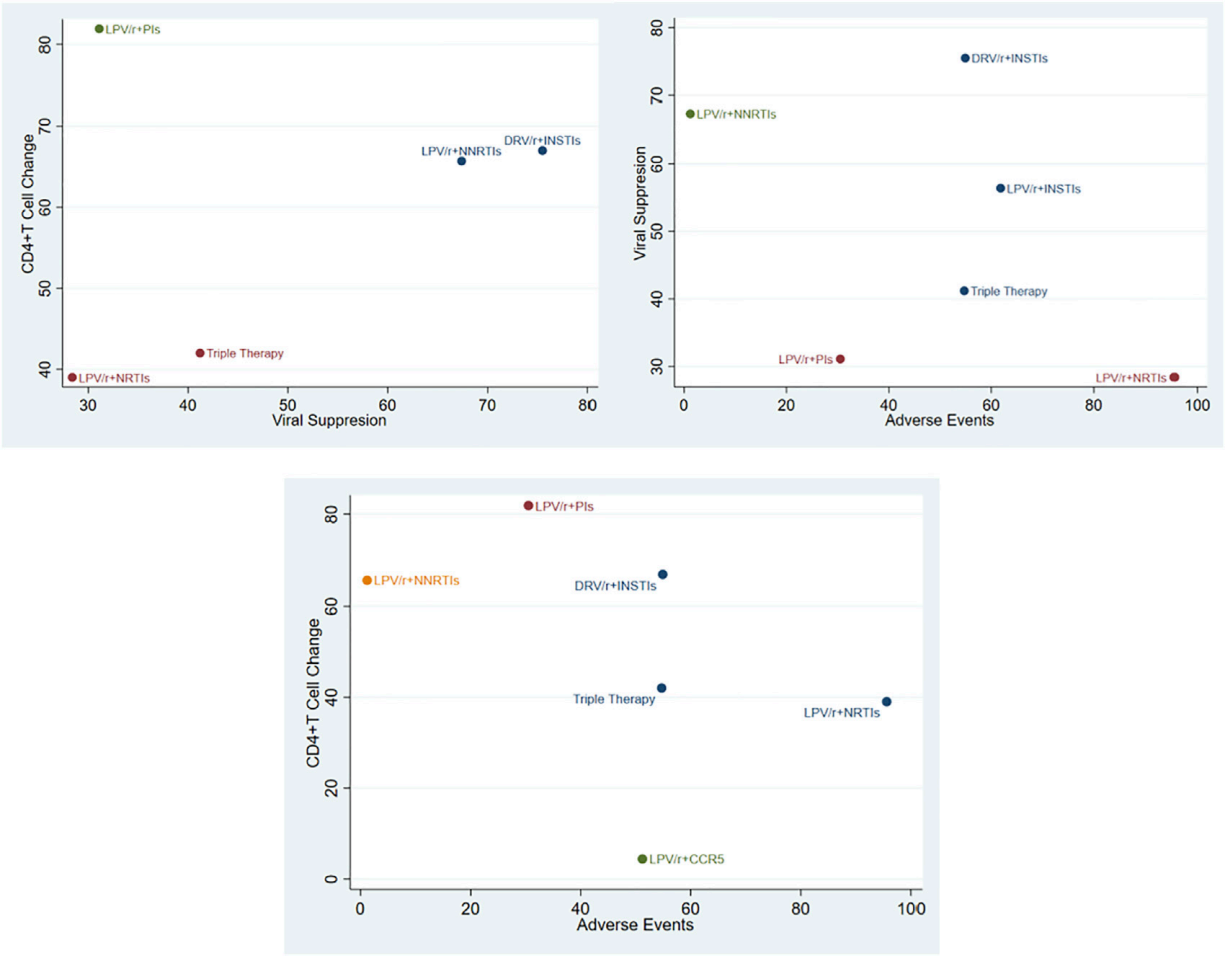
Cluster diagram among different outcome indicators.

The viral suppression rate, the incidence of adverse events and the number of CD4^+^T cell changes were analyzed by pairwise clustering (Figure 5). It can be seen that DRV/r + INSTIs was superior to TT in inhibiting HIV, improving immune reconstruction. The incidence of adverse events was equivalent to TT, which was consistent with the results of forest map and SUCRA ranking chart.

## DISCUSSION

Our study compared the different interventions for treatment-naïve, HIV/AIDS patients. As a statistical method of indirect comparison through direct comparison, the network meta-analysis compares the advantages and disadvantages of different interventions and ranks them to screen the best intervention (Buti et al., 2011). PIs/r was suggested as an attainable maintenance strategy in patients achieving stable HIV suppression in plasma (Pinnetti et al., 2014), which mainly contains DRV/r and LPV/r. Many studies (Bedimo et al., 2014; Di Cristo et al., 2020) have confirmed that DRV/r-based dual simplified therapy could be regarded as an alternative treatment for naïve AIDS patients. Since LPV/r is not a component of the first-line antiviral scheme, LPV/r based dual therapy is not recommended in most guidelines. However, LPV/r is widely used in China, because it is incorporated into free drug catalogues. If DRV/r is hard to achieve, LPV/r could be used as an alternative scheme. For the efficacy of different interventions, it is crucial to evaluate the index of viral suppression, △CD4^+^T cell changes from baseline, adverse events. Our results showed that PIs/r based on DT was significantly better than traditional TT, which was consistently with the reported studies. Huang et al. (Huang et al., 2019) compared the efficacy and safety of RAL based DT in AIDS patients. They found that the simplified DT regimen brought a better CD4^+^T cell count and lower rate of adverse events than the TT regimen.

We also sorted the different interventions by the SUCRA values, which was consistent with the results of forest map. We found that DRV/r + INSTIs showed significant advantages among the different schemes, which could not only inhibit HIV replication, but also significantly improve immune reconstruction. It was found that DRV/r + INSTIs was equivalent to TT in terms of adverse reactions. Therefore, it was reasonable to think that DRV/r + INSTIs could be used as one of the effective alternatives of TT.

Recently, many studies have focused on DRV/r + INSTIs (Calza et al., 2020; Fokam et al., 2020). Stellbrink et al. (Stellbrink et al., 2016) suggested that DRV/r + INSTIs was more effective than TT in increasing body fat and improving physical fitness. Compared with TT scheme, DRV/r + INSTIs may have less effect on bone and does not affect the synthesis of 25(OH)_2_D_3_, and the economic burden of DRV/r + INSTIs is obviously lower than TT. A cohort study from France showed (Cahn et al., 2014) that the efficacy of DRV/r + INSTIs and ATV/r was compared in naïve HIV/AIDS patients with severe immunosuppression, based on the original treatment scheme. It was also found that there was no significant difference between DRV/r + INSTIs and ATV/r in inhibiting virus and increasing CD4^+^T cells. Another study (Bedimo et al., 2014) compared the insulin sensitivity between DRV/r + INSTIs and ATV/r which had the same effect on blood glucose, however, there was no significant difference in insulin sensitivity. In addition, a major challenge in HIV/AIDS treatment is polypharmacy and, consequently, drug–drug interactions (DIs). Pontelo et al. (Pontelo et al., 2020) pointed that PI-based antiretrovirals (ARVs) regimen were independently associated with DIs. The same finding was reported by Farhoudi et al. (Farhoudi et al., 2015). ATV-containing ARVs was the regimens with more DIs clinically significant (71%), DRV-based regimens presented a little of clinically significant (15%). Moreover, ATV is not among the first choices for PI-based ARV regimens. In all, it is worth regarding that DRV/r + INSTIs is a recommended treatment scheme in terms of efficacy and safety.

The treatment-naïve, HIV/AIDS patients in our study are supported by the following factors. Firstly, treatment-naïve HIV/AIDS patients have better sensibility to drugs, who are the most ideal object for achieving viral suppression. Secondly, the selection of treatment plan for naïve HIV/AIDS patients needs comprehensive evaluation of various factors, including age, co-infection, CD4^+^T cell counts, viral load and drug side effects. In addition, the economic pressure should be considered. Vizcarra et al. (Vizcarra et al., 2019) found that dual therapy can obviously reduce the burden of patients. Finally, it is necessary for naïve AIDS patients to choose the appreciate treatment plan to reduce changing medicine and interrupting disable easily. Notably, patient compliance and drug adherence can significantly hamper effectiveness of the PIs/r-based treatment regimen (Squires et al., 2016). It is necessary to take this issue into account when PIs/r-based drug combination is carried out, which provide reference for more related researches.

There are some limitations in this study: 1) The number of individual intervention measures or outcome indicators included in this study is small, and there may be some publication bias; 2) In terms of literature quality evaluation, most of the studies are open label research, and the random methods of some studies are not clearly described, which may bring distribution bias to a certain extent; 3) There are some differences in the TT schemes included in the study, which may affect the results.

## Conclusion

PIs/r-based dual simplified therapy can be used as a reliable simplified scheme for naïve AIDS patients. Among them, DRV/r + INSTIs dual simplified scheme had remarkable effectiveness in inhibiting HIV replication and immune reconstruction, and was expected to become an effective alternative to triple scheme.

## Data Availability

The original contributions presented in the study are included in the article/Supplementary Material, further inquiries can be directed to the corresponding author.
