# Supplementary material for: Effectiveness and Safety Analysis of PIs/r Based Dual Therapy in Treatment-Naïve, HIV/AIDS Patients: A Network Meta Analysis of Randomized Controlled Trials
Source: Front Pharmacol. 2022 Mar 4;13:811357. doi: 10.3389/fphar.2022.811357 (PMC8931831; doi:10.3389/fphar.2022.811357)
Supplement: Supplementary file 1 [file DataSheet1.docx]

Supplementary Material

# Supplementary Data

Supplementary Material should be uploaded separately on submission. Please include any supplementary data, figures and/or tables. All supplementary files are deposited to FigShare for permanent storage and receive a DOI.

Supplementary material is not typeset so please ensure that all information is clearly presented, the appropriate caption is included in the file and not in the manuscript, and that the style conforms to the rest of the article. To avoid discrepancies between the published article and the supplementary material, please do not add the title, author list, affiliations or correspondence in the supplementary files.

# Supplementary Figures and Tables

## Supplementary Figures


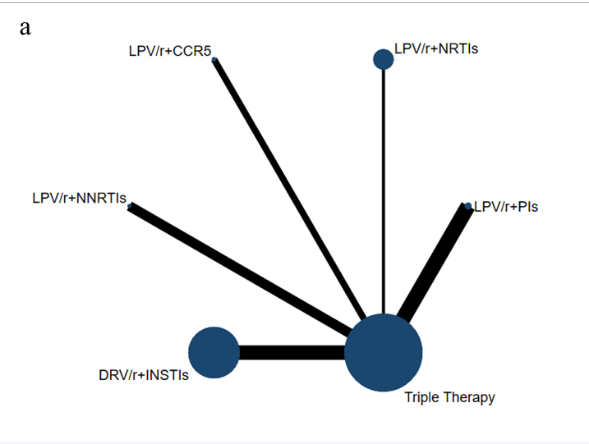

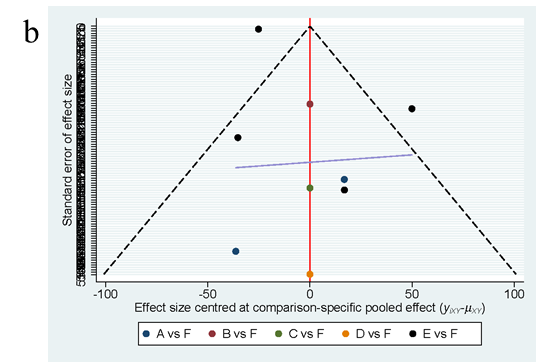


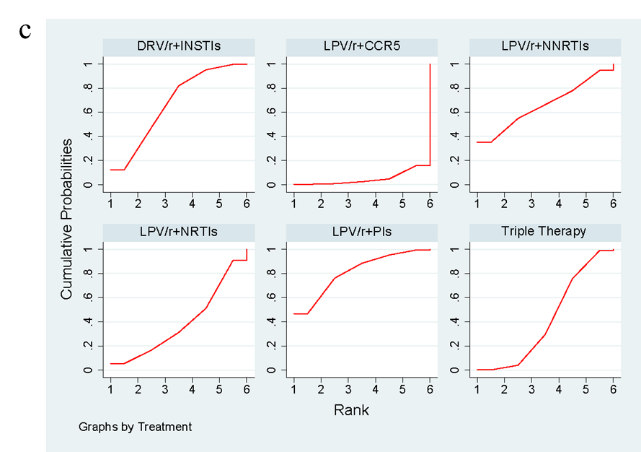


**Supplementary Figure S1.** The Results of Network Meta-analysis with CD4^+^T cell count change from baseline. a: The network diagram. b: The comparison-correction funnel diagram (A: LPV/r + PIs, B: LPV/r + NRTIs, C: LPV/r + CCR5, D: LPV/r + NNRTIs, E: DRV/r + INSTIs, F: Triple Therapy). c: SUCRA value ranking chart.


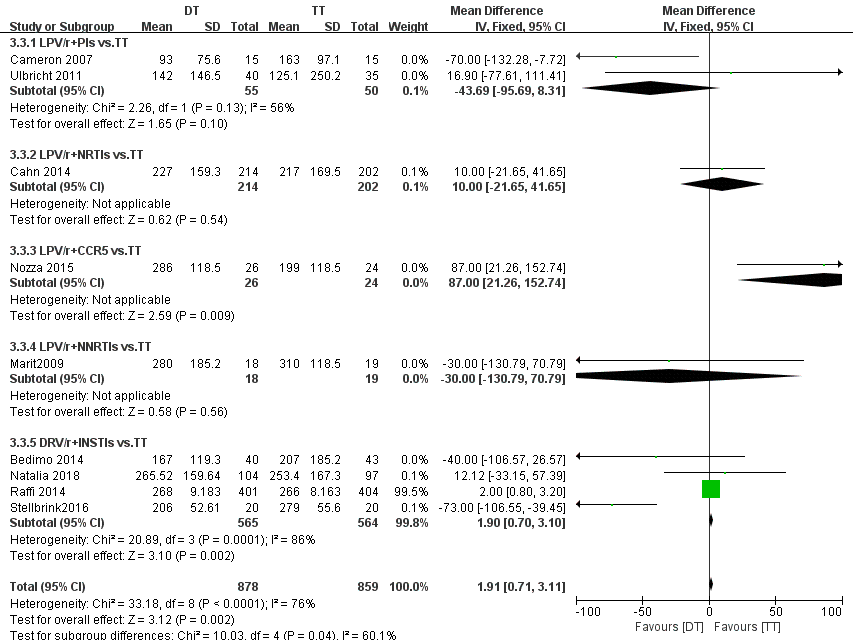


**Supplementary Figure S2.** Node-splitting of Network Meta-analysis Based on CD4+T cell count change from baseline.


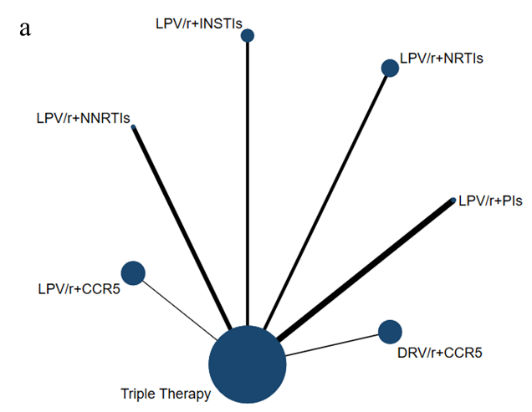

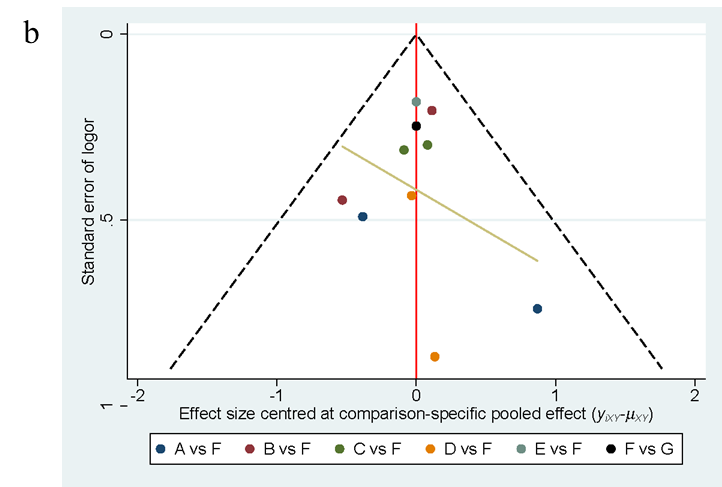


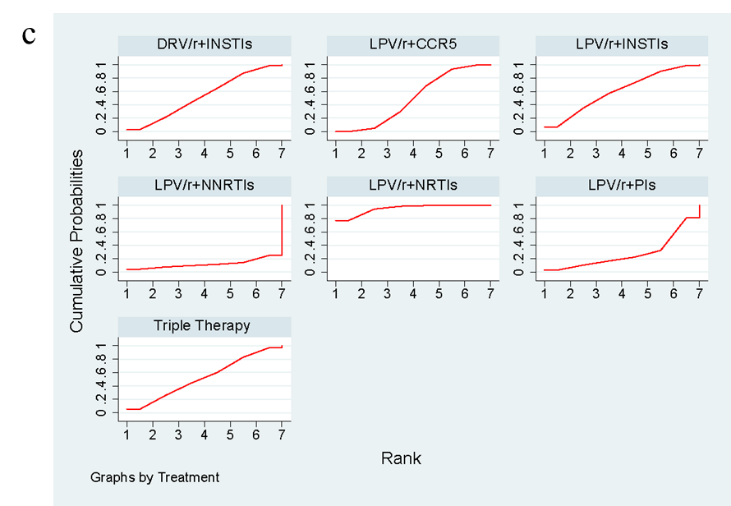


**Supplementary Figure S3.** The Results of Network Meta-analysis with Adverse Events Rate. a: The network diagram. b: The comparison-correction funnel diagram (A: LPV/r + PIs, B: LPV/r + NRTIs, C: LPV/r + INSTIs, D: DRV/r + INSTIs, E: LPV/r + CCR5, F: Triple Therapy, G: LPV/r + NNRTIs). c: SUCRA value ranking chart.


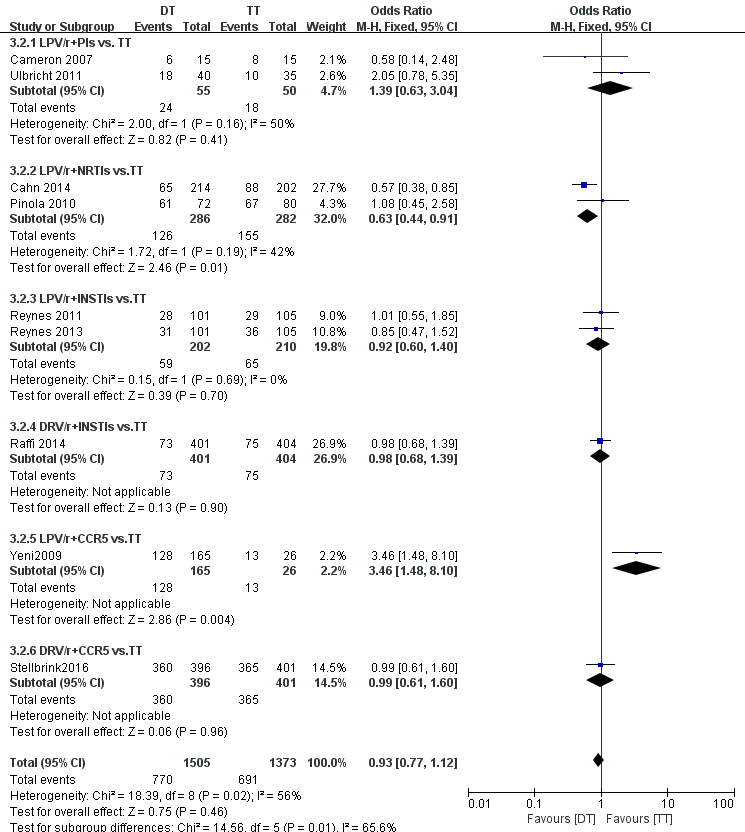


**Supplementary Figure S4.** Node-splitting of Network Meta-analysis Based on Adverse Events.
